# Supplementary material for: Exploring TRF2-Dependent DNA Distortion Through Single-DNA Manipulation Studies
Source: Commun Biol. 2024 Feb 3;7:148. doi: 10.1038/s42003-024-05838-x (PMC10838314; doi:10.1038/s42003-024-05838-x)
Supplement: Supplementary file 2 — Supplementary Information [file 42003_2024_5838_MOESM2_ESM.pdf]

Supplementary Information of "Exploring TRF2-Dependent DNA Distortion Through Single-DNA Manipulation Studies"

SUPPLEMENTARY INFORMATION

Supplementary Information 1 for "Multiple DNA binding modes of TRF2 are revealed by single-DNA manipulation"

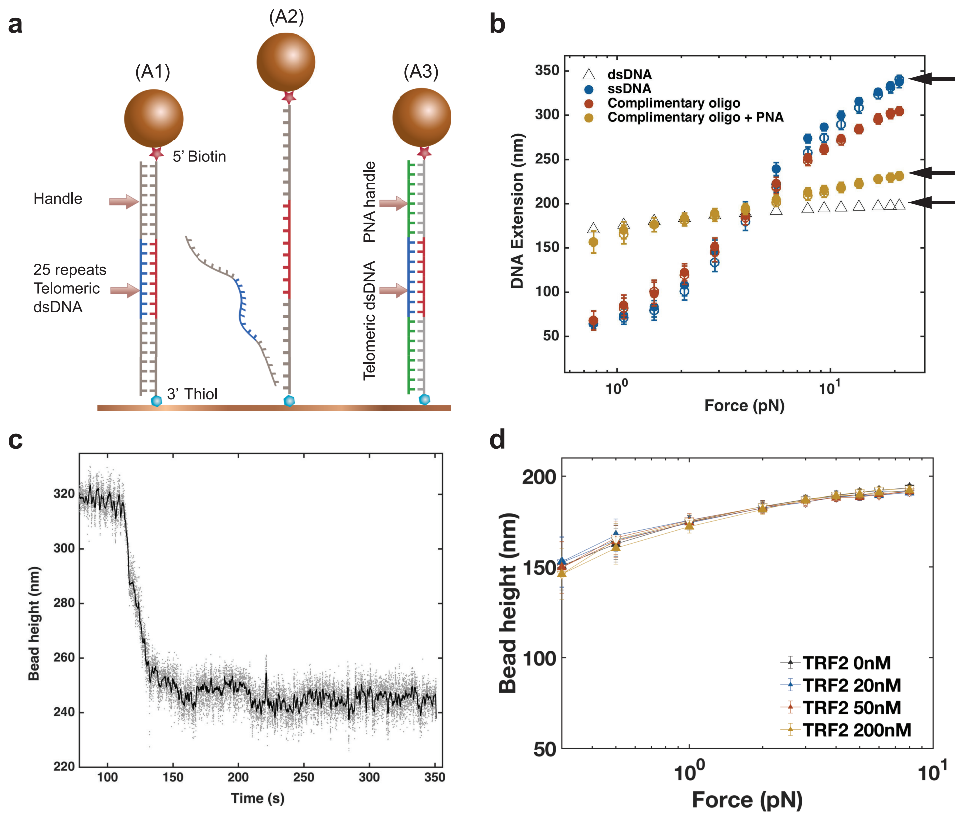

Supplementary Figure 1. Single-DNA stretching and hairping assays. **a** Schematic of building DNA template composed of 25 repeats of telomeric DNA and two DNA handles labelled with 5′ biotin and 3′ thoil on the same strand in three steps. Starting from a dsDNA (A1), an ssDNA is generated by force-dependent strand-peeling transition (A2). Finally, a complementary strand is flowed in to form a specific dsDNA binding site and complementary PNA oligos were added to block the ssDNA overhangs (A3). **b** Force-extension curves of sequential steps of building site-specific telomeric DNA from initial dsDNA (A1) to ssDNA (A2), followed by adding complementary ssDNA and PNA oligos (A3). **c** Bead height decreases as PNA oligos annealing to the ssDNA handles at $\sim$ 20 pN. **d** No apparent deformation for TRF2 binding to 600 bp PNA/ssDNA up to 200 nM. The error bars in b and d are standard deviations of the bead height during the recording time window.

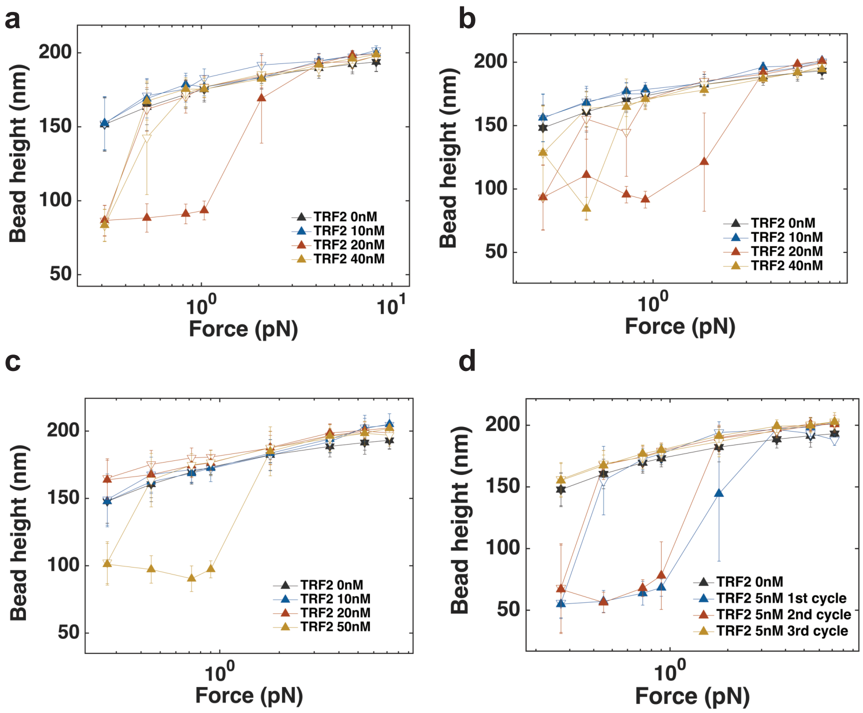

Supplementary Figure 2. DNA force-height curves obtained at different TRF2 concentrations a-d Additional DNA force-height curves were obtained in a force-decrease scan (hollow symbols) followed by a force-increase scan (solid symbols) at several different TRF2 concentrations. **d** The hyteresis between the force-decrease and the subsequent force-increase curves was observed in the first two force-decrease and force-increase cycles and it disappeared in the third cycle. This is consistent with the slow rate for the transition to the more stable compact conformation. The error bars are standard deviations of the bead height during the recording time window.

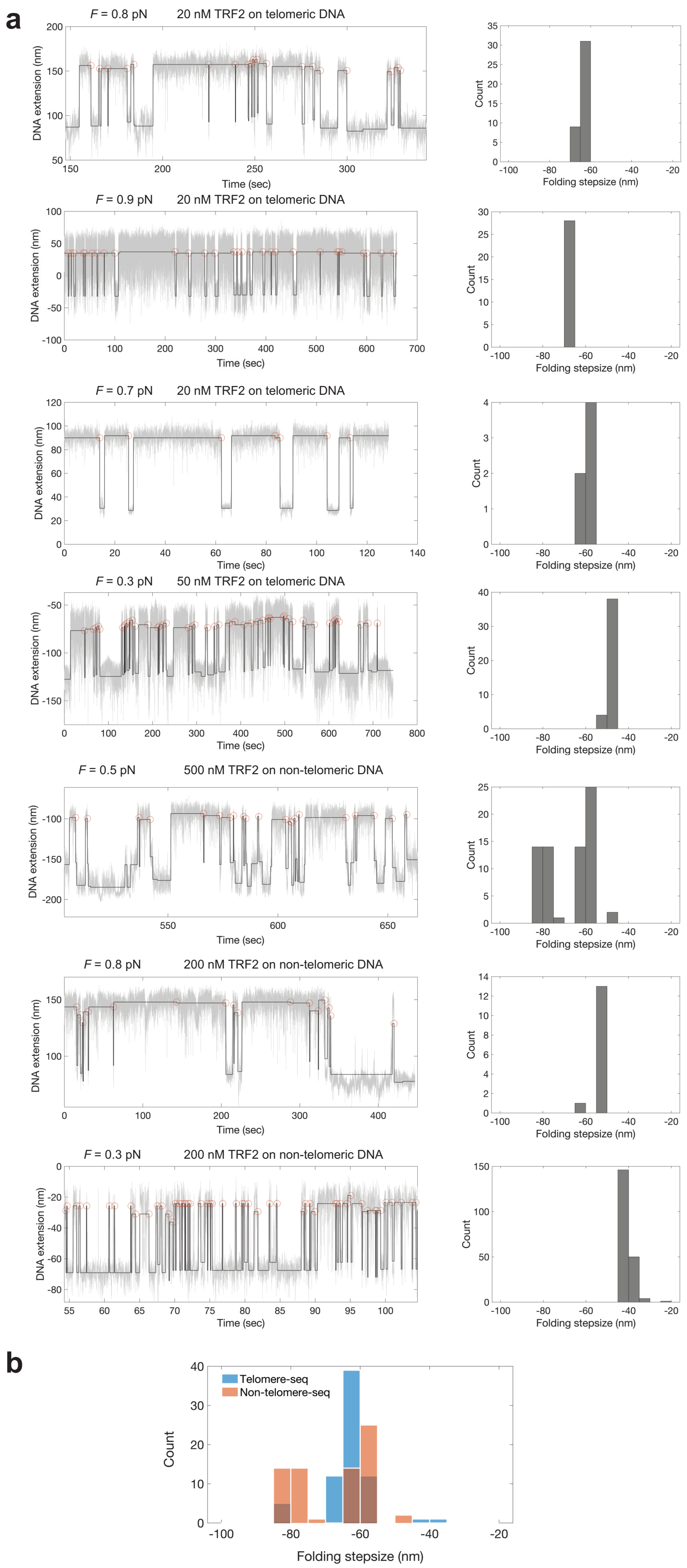

Supplementary Figure 3. Step-sizes for the transitions between extended and compact DNA. a Seven representative time traces of bead height in TRF2 solution from independent DNA tethers (left), and the corresponding histrograms (right). **b** Histogram of step-sizes from all telomeric and non-telomeric DNA (N=7).

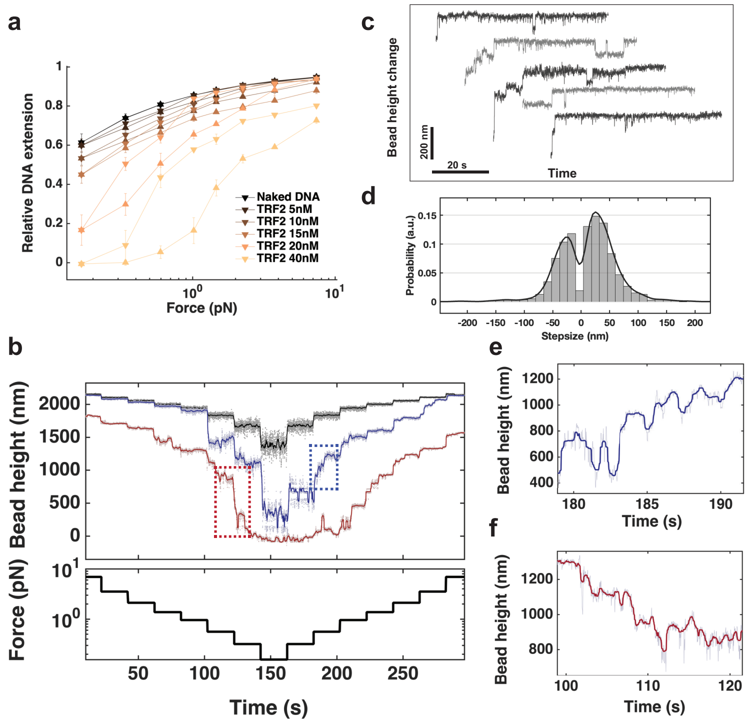

Supplementary Figure 4. TRF2 binding to longer non-telomere dsDNA **a** Force-height curves of a 6.5 kbp non-telomeric DNA molecule at varying concentrations of TRF2 are shown. At each force, the tether was held for 20 seconds, and the average extension was recorded. The force-height curve for bare DNA is represented by black color and recorded in both force-decrease scans (solid down-triangles) and force-increase scans (solid up-triangles). As the TRF2 concentration increases (shown in sequential decrease in brown color), the level of DNA compaction also increases, and hysteresis between the force-decrease and force-increase curves becomes apparent. Furthermore, even at the highest force (8 pN), the DNA can still remain partially compacted, indicating the formation of TRF2 nucleoprotein complexes with different stabilities. The error bars are standard deviations of the bead height during the recording time window. **b** Stepwise folding and unfolding events are observed in real-time force-decrease and force-increase curves at each constant force during force scanning below 2 pN (zoom in E-F). This suggests that TRF2 DNA folding units, similar to those observed on short DNA molecules (Fig. 2 in the main text), also form on the larger DNA molecule. **c** Representative time traces of folding and unfolding events are shown as the applied force jumps from 0.5 pN to 1.5 pN in the presence of 20 nM TRF2. **d** The step sizes were fitted using Hidden Markov Models in Matlab and plotted as a histogram. The peak locations of -27.5 nm and 25.2 nm were estimated based on a normal kernel function in Matlab. A total of 3844 steps were collected from 15 individual longer DNA tethers. **e-f** Zoomed-in time traces, marked as rectangles in (B), provide a closer look at the folding and unfolding events.

Supplementary Information 2 for "TRF2 binding does not have strong supercoiling chirality preference"

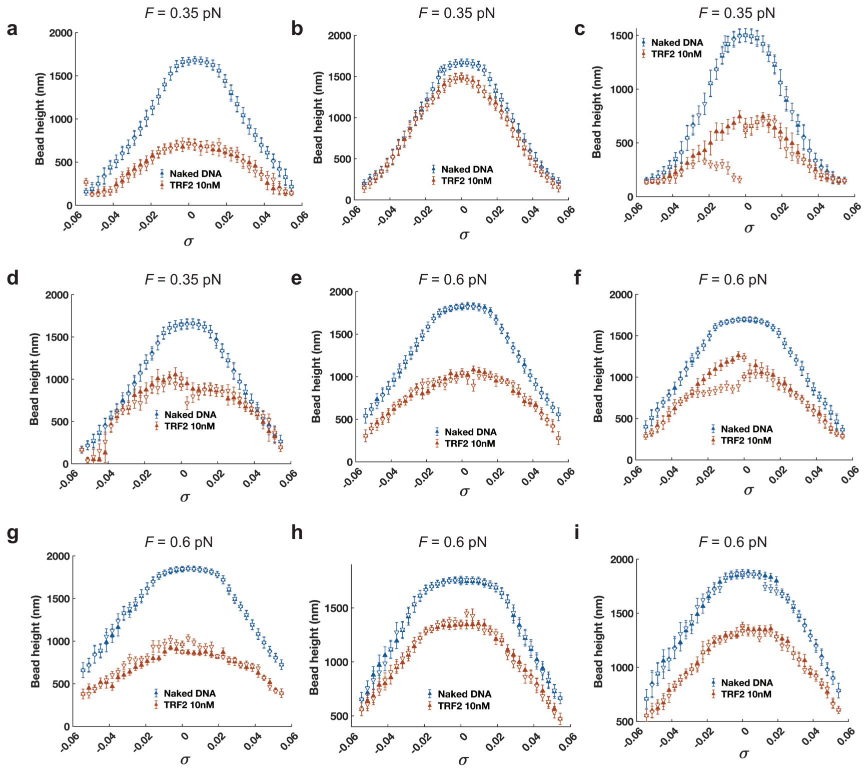

Supplementary Figure 5. TRF2 binding to supercoiling dsDNA in an extended time scale a-i σ-H curves obtained for TRF2-DNA interaction with 60 seconds sampling duration for each data point. The total duration is almost three hours to record one σ-H curve. The resulting σ-H curves show qualitatively similarity to the curves in the main text (Fig. 3b-c) obtained with 10 seconds sampling duration. The error bars are standard deviations of the bead height during the recording time window.

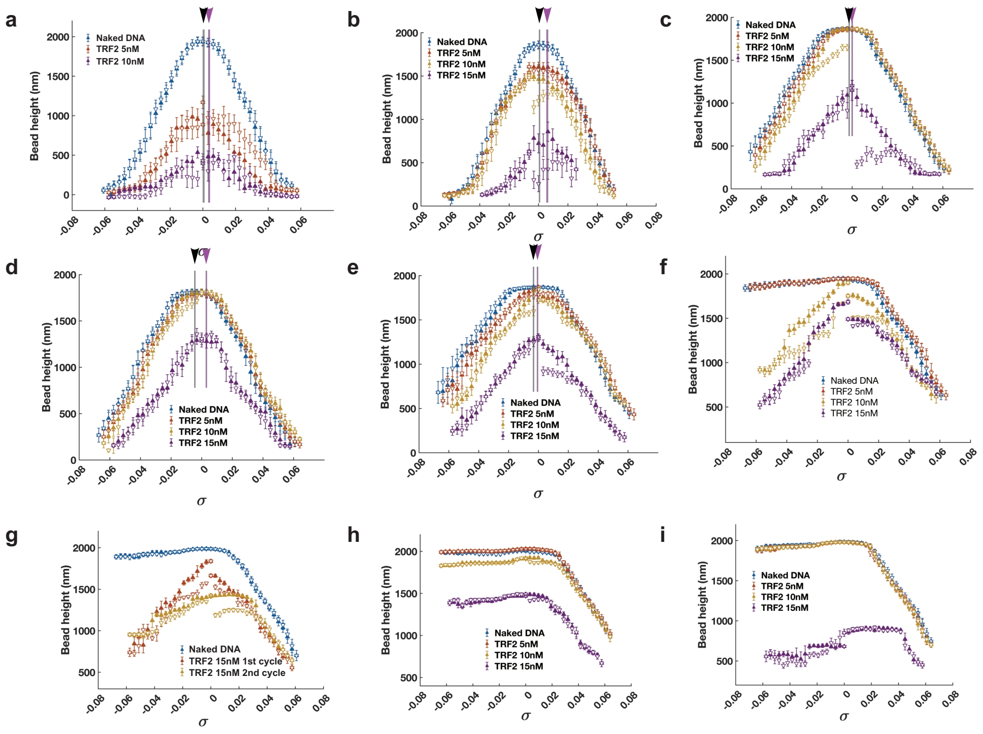

Supplementary Figure 6. Additional examples of TRF2 binding to supercoiled dsDNA at different forces a-i In the majority of experiments, nine of twelve independent experiments including nine in the supplementary material and three in the main text, the effect of TRF2 on DNA supercoiling under force is consistent with the picture that TRF2 bends DNA and facilitates both (+) and (-) DNA supercoiling by reducing the energy barrier of DNA buckling transition. In panels (A-E), the black and purple downward arrows indicate the apparent supercoiling center in the absence of TRF2 and with the presence of 15 nM TRF2, respectively. The error bars are standard deviations of the bead height during the recording time window.

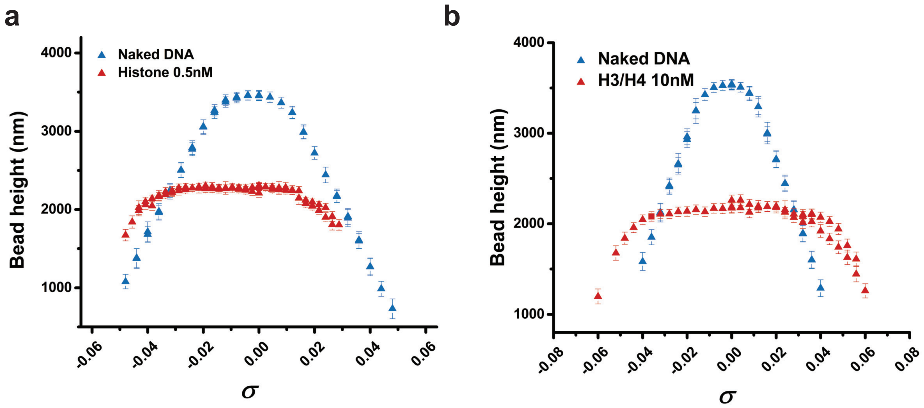

Supplementary Figure 7. Supercoiling assays of histone octamer and histone tetramer Representative bead height as a function of DNA supercoiling density changes at 0.45 pN constant force. **a** Histone octamer assembly on 13-kbp linking number-constrained DNA results in broadening of the σ-H curve and shifting the supercoiling center to the left. This observation is consistent with left-handled wrapping of histone octamer binding to DNA. **b** Histone tetramer assembly on the same DNA only results in broadening of the σ-H curve without supercoiling center shift. The feature of such σ-H curve suggests that histone tetramer causes DNA wrapping without significant chirality preference. The error bars are standard deviations of the bead height during the recording time window.

Time dependent of TRF2 binding on supercoiled DNA

Regarding whether the more stable TRF2-DNA complex can wrap DNA with chiral preference, we have conducted new supercoiling experiments over different time scales at 60, 300, 600 seconds. For each experiment, the torque constraint DNA tethers were first held at 0.3 pN and twisted to $\Delta Lk = +20$ (Fig.S 8A) to form positive supercoiled DNA and to $\Delta Lk = -20$ to form negative supercoiled DNA, respectively. The formed supercoiled DNA remained for designed time windows (60, 300, 600 seconds). After that, the tethered DNA was twisted back to $\Delta Lk = 0$. In the presence of TRF2 20 nM, slower relaxation and shorter extension of bead height was observed in Fig.S 8B compared to sharp bead extension jump in Fig.S 8A. Data points marked in red represent a 60-second time trace of bead height immediately after twisting the DNA from Lk= +20 or Lk= -20 back to Lk= 0. The mean value of the bead height during this time window is summarized in Fig.S 8C, revealing no detectable chiral preference of DNA binding by TRF2.

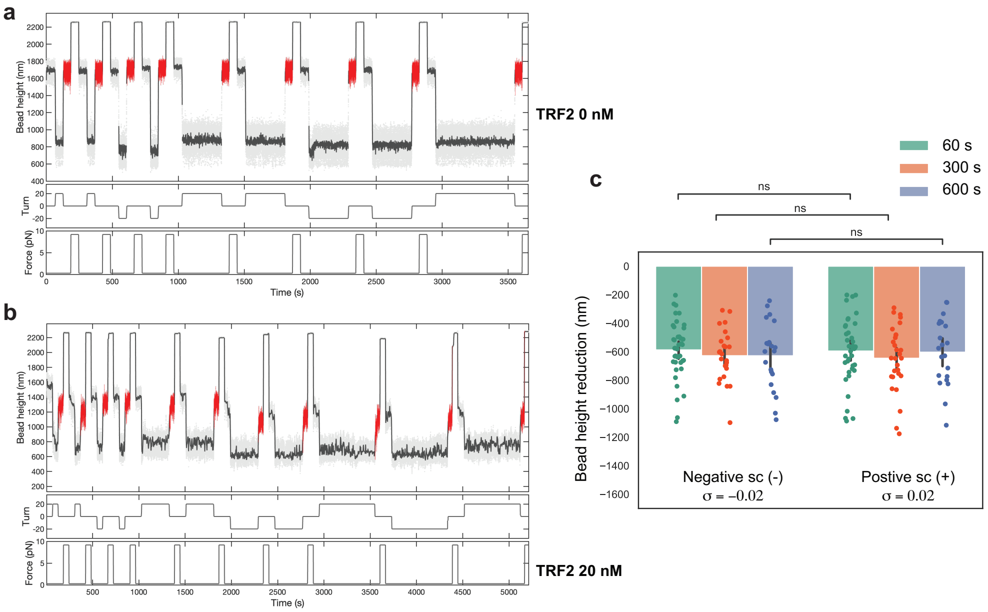

Supplementary Figure 8. Time dependent of TRF2 binding on supercoiled DNA. The top panels are representative time traces depicting the change in bead height resulting from the winding of DNA 20 turns (middle panels) to form positive supercoiled DNA at $\sigma = 0.02$, with the tether held at 0.3 pN. These traces illustrate the absence of TRF2 (**a**) and the presence of 20 nM TRF2 (**b**). Following the formation of positive supercoiled DNA, it was maintained for 60 seconds before being relaxed to $\sigma = 0$. The duration of supercoiled DNA state remained for 300 and 600 seconds were shown on subsequent cycles. To prepare for the subsequent twisting cycle, the force was abruptly increased to 30 pN (bottom panels) in order to remove the bound TRF2 in the case of the presence of proteins. **c** The Mann-Whitney U test is used to compare TRF2 binding induced bead height deduction between positive supercoiled DNA and negative supercoiled DNA. The p-values are greater than 0.05, considered statistically insignificant, for all time scales at 60, 300, 600 seconds.

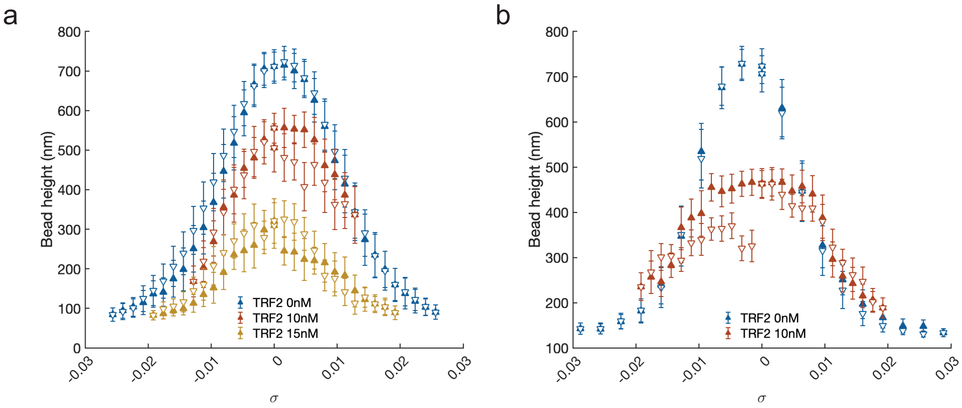

Supplementary Figure 9. TRF2 binding to supercoiled dsDNA containing 25 telomeric DNA repeats a-b Two representative bead height (H) as a function of DNA superhelical density (σ) changes when DNA is held at 0.35 pN. A 3-kb DNA fragment (PUC57) containing 25 repeats of TTAGGG was wound/unwound to generate positive/negative supercoiled DNA at approximately 0.3 pN. In the presence of TRF2, the decrease in bead height indicates protein-induced compaction upon binding. The absence of a shift in the curve peak suggests no significant chiral binding of TRF2 to the inserted telomere sequence. The error bars are standard deviations of the bead height during the recording time window.

Alternative method 1 to test chirality preference of TRF2 binding to DNA

The torque constraint DNA molecule was conducted a (+) or (-) linking number change at a linking number density of ±0.03, while maintaining a fixed force of 0.3 pN to allow the formation of supercoiled plectonemes. It followed by introducing 20 nM TRF2 to observe any binding-induced changes in bead height (Fig.S 10). What we observed is that TRF2 induced further compaction of the DNA, regardless whether it was wound(+) or underwound(-). These findings indicate that TRF2 binding does not induce significant preferential chiral deformation of DNA, as there were no TRF2-induced significant relaxation of DNA supercoiling changes observed for both (+) and (-) supercoiled plectonemes. In this assay, to prevent any drag force from being applied to the DNA during the introduction of TRF2 solution, we utilized a PDMS membrane well-based perturbation isolation technology [1].

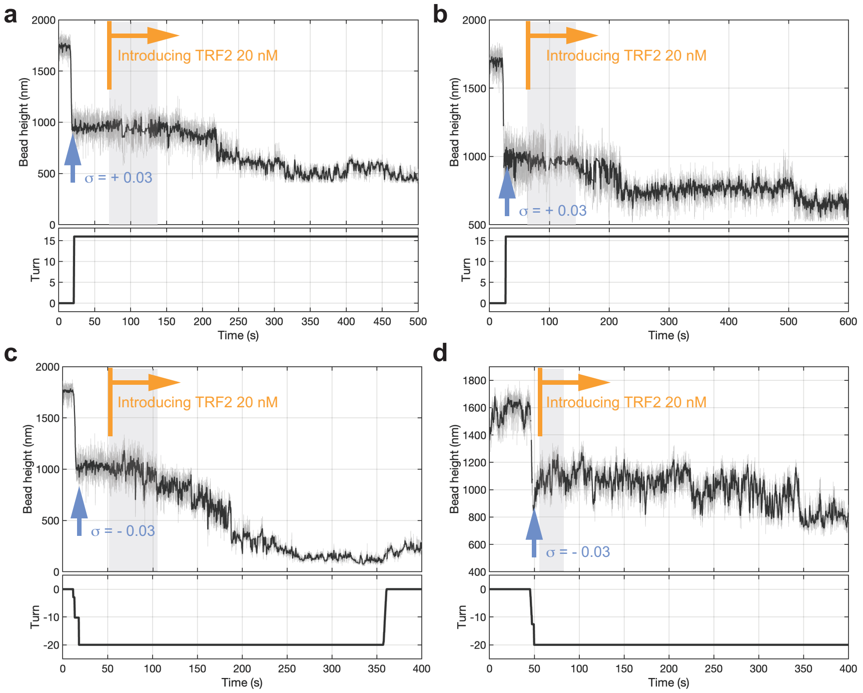

Supplementary Figure 10. TRF2 binding on pre-formed supercoilling DNA Representative time traces of TRF2 binding on positive supercoiling DNA (**a-b**) and negative supercoiling DNA (**c-d**). The shaded regions indicate the buffer exchange process for 20 nM TRF2.

Alternative method 2 to test chirality preference of TRF2 binding to DNA

A different approach to test chirality preference of TRF2 binding to DNA is shown as following. Initially, we held a DNA at zero linking number density at 0.3 pN and recorded the bead height. We then introduced 20 nM TRF2 with the perturbation isolation system and recorded the further reduction in the bead height caused by TRF2 binding. If TRF2 induces preferential chiral wrapping at its binding sites, then at TRF2-free regions, the DNA should form plectonemes with an opposite chirality to ensure unchanged linking number of the DNA.

With this background, if we introduce opposite winding to DNA, the plectonemes in the TRF2-free region should be relaxed (an effect similar to introducing TOP1), resulting in increased DNA extension. However, we did not observe any extension increases after the introduction of TOP1. Fig.S 11A shows the extension change of a DNA tether before and after introduction of 20 nM TRF2 at zero linking number change. DNA becomes shorter and exhibited dynamic fluctuation, indicating TRF2 mediated dynamic compaction of DNA. At time of 2000 s, the DNA was subjected to unwinding of -10 turns (Lk=-10), followed by winding of +20 turns (Lk=+10), and then unwinding of -10 turns (Lk=0). As shown in the zoom-in in Fig.S 11B, the extensions at Lk=+10 and Lk=-10 are both not longer than the extension before the winding and unwinding processes. The results indicate that both winding and unwinding produced further plectonemes with the corresponding chirality, which are then stabilized by TRF2. Fig.S 11A and C show data repeated on a different DNA tether. Together, these observations are consistent with no strong chiral deformation of DNA by TRF2 binding.

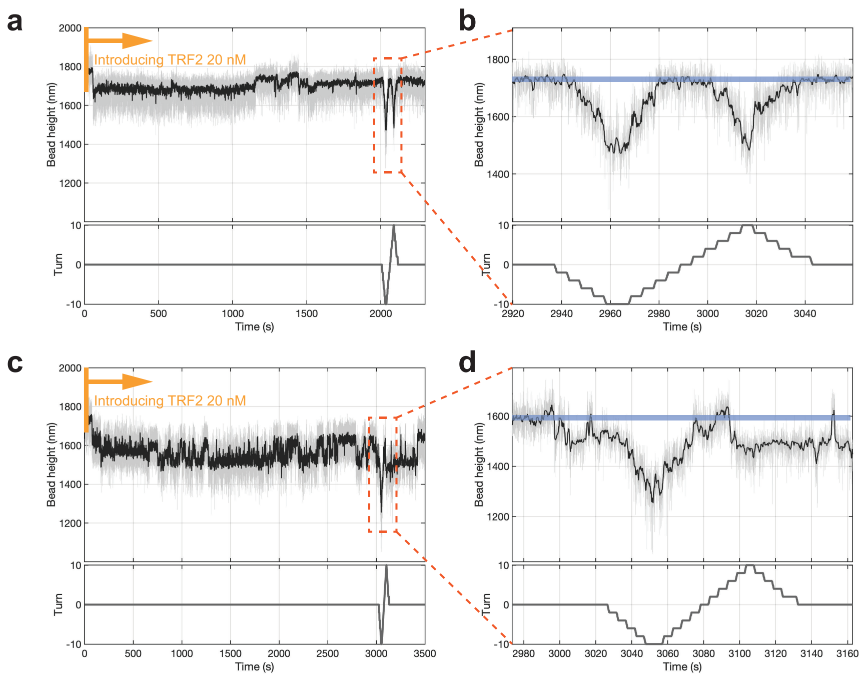

Supplementary Figure 11. Detection chirality preference binding of TRF2 by winding/unwinding DNA (**a, c**) Representative time traces after adding 20 nM TRF2 and incubating for approximately 3000 seconds. Subsequently, the DNA was unwound and rewound (ΔLk shown in the bottom panels), and the change in DNA extension was examined before and after this process. (**b, d**) Zoomed-in time traces are displayed, corresponding to the red rectangles marked in panels A and C.

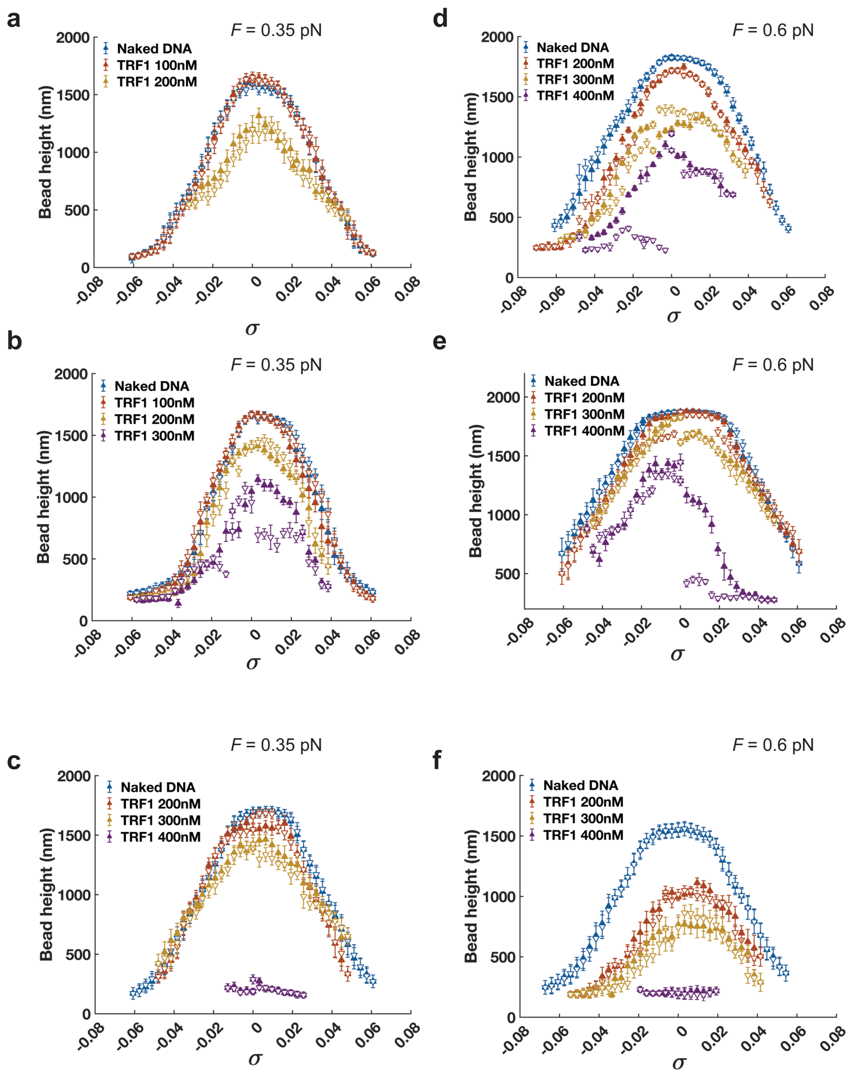

Supplementary Figure 12. single-DN supercoiling assay for TRF1. **a-f** Representative σ-H curves shows that TRF2 binding resulted in narrower parabolic caps without changing the center of the curves, a characteristic of DNA-bending proteins. The error bars are standard deviations of the bead height during the recording time window.

Supplementary Information 3 for "The determinants of TRF2-DNA binding affinity"

PNA/ssDNA effectively suppresses TRF2's branch-binding

Supplementary Figure 13a is schematic of building a DNA hairpin composed of 5 repeats of telomeric DNA stem and two PNA DNA handles. The inverted repeats indicated by blue and red colours on the tethered strand is ligated with two handles. After remove the complementary strand, a DNA hairpin flanked by two ssDNA handles is formed at forces below the unzipping force. We demonstrated that using ssDNA handles to span the same hairpin resulted in strong TRF2 binding that completely prevented the unzipping of the DNA hairpin at the probing force Supplementary Figure 13d. This indicates TRF2 has a strong affinity with the fork between the dsDNA hairpin and the spanning ssDNA handles, consistent with previous biochemical studies [2]. This is in sharp contrast to the transient pauses prior to DNA unzipping observed during force jumping from F_b to F_p when using PNA/ssDNA handles Supplementary Figure 13c. This result provides additional evidence that the PNA/ssDNA effectively suppresses TRF2's branch-binding. This suppression can be attributed to the non-native conformation of the PNA/ssDNA handles, which is unfavorable for TRF2 binding. Furthermore, we conducted experiments using 1000 nt ssDNA and observed a distinct TRF2-dependent shift in the force-extension curve (Supplementary Figure 13b). This finding indicates the presence of an ssDNA-binding activity of TRF2, which can account for the branch-binding activity of TRF2 when the hairpin is bridged between ssDNA handles.

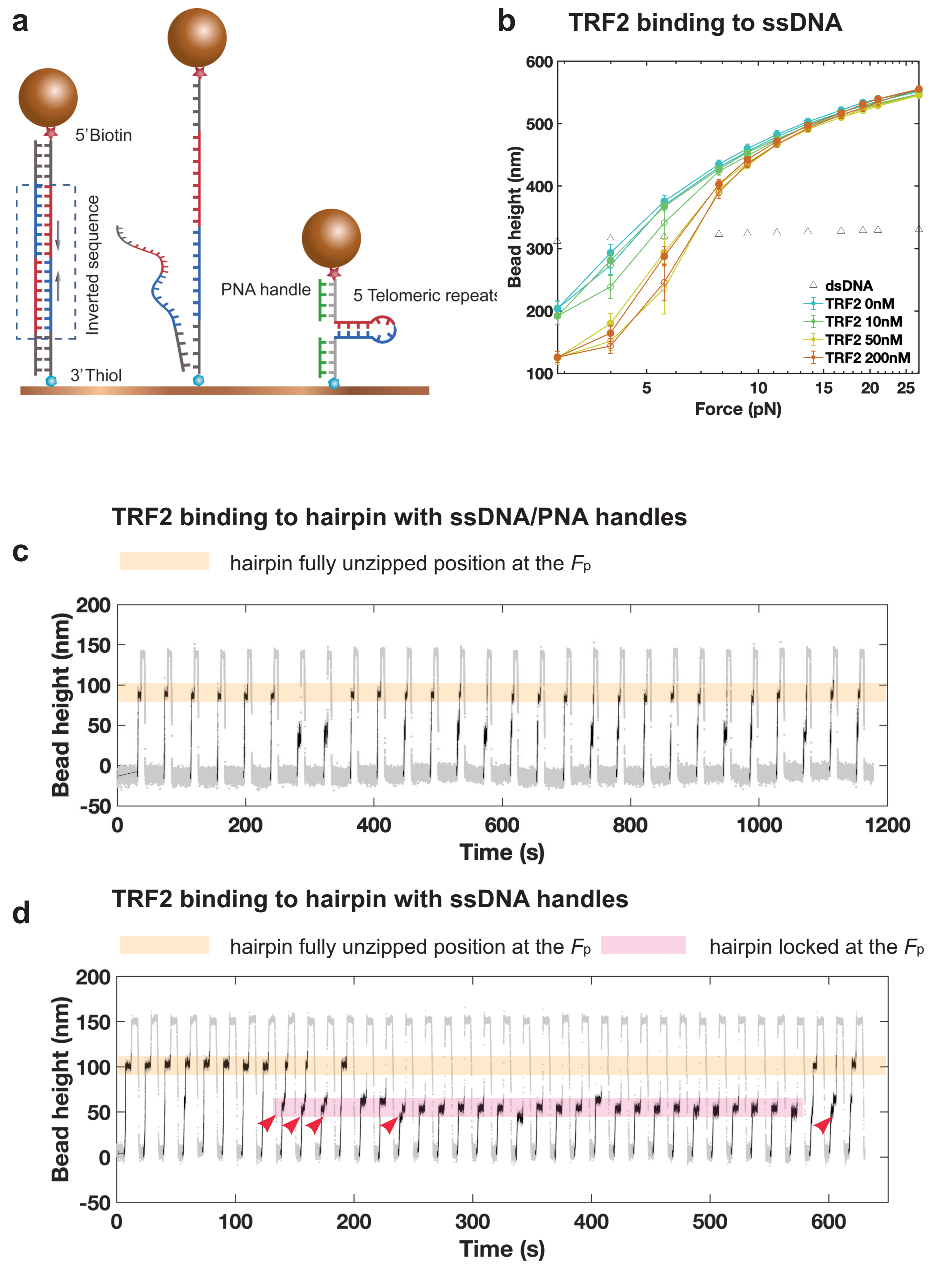

Supplementary Figure 13. PNA/ssDNA effectively suppresses TRF2's branch-binding. **a** Schematic of integrating inverted sequence to build DNA hairpin composed of 5 repeats of telomeric DNA stem and two DNA handles labelled with 5′ biotin and 3′ thoil on the same strand in three steps. **b** TRF2 compact 1000 nt ssDNA as concentration increasing to 50 nM. The error bars are standard deviations of the bead height during the recording time window. Representative time trace for TRF2 binding on DNA hairpin flanked by PNA/ssDNA duplex handles (**c**) and ssDNA handles (**d**), respectively.

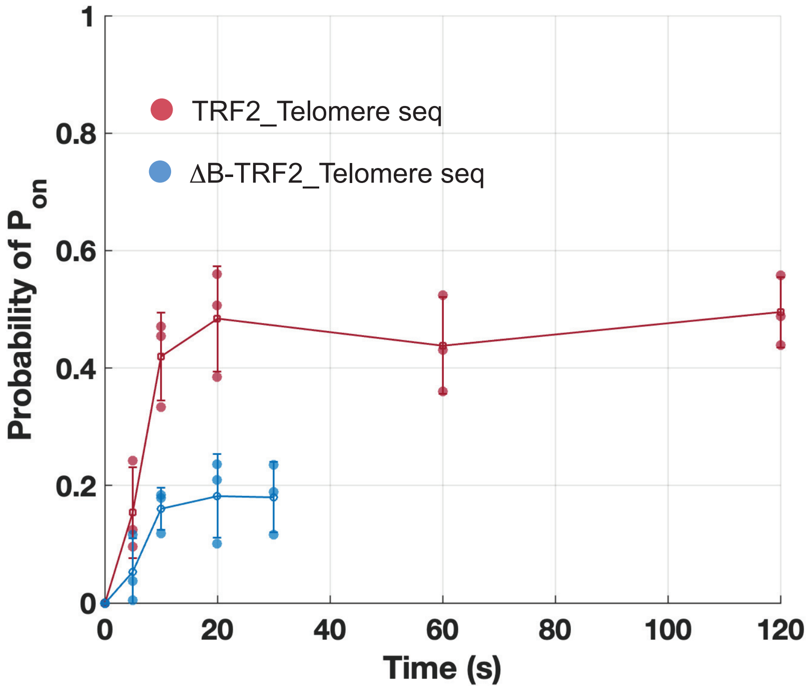

Supplementary Figure 14. The time-dependent probabilities of binding $p(\Delta T)$ The binding probability curves curve reaches a plateau after incubation time above 10 seconds for both TRF2 and ΔBTRF2, suggesting that binding probability reach equilibrium after 10 seconds incubation time. The error bars represent the standard deviation of independent experimental data.

Bootstrap analysis

In the measurement of the equilibrium binding probability at different TRF2 concentrations, a repetitive force-jump procedure was employed. For each DNA tether, the force-jump procedure was repeated for 50 times, from which a probability of binding was estimated based on the fraction of the cycles where TRF2 binding was detected. At each TRF2 concentration, such measurement was repeated for five independent tethers, from which a mean μ and a standard error of the mean σ were obtained. Assuming the uncertainty of the mean described by Gaussian distribution, the standard error of the mean can be interpreted to be the standard deviation of the probability of the mean. Therefore, the distribution of the mean of the binding probability $\bar{p}$ can be written as: $\rho(\bar{p}) = \frac{1}{2\pi\sigma^2} e^{\frac{(\bar{p}-\mu)^2}{2\sigma^2}}$. Based on this distribution, we resampled the mean of probability by 1000 times and use data in $-2\sigma \leq \bar{p} \leq 2\sigma$ to obtain the sampled mean and sampled standard deviation.

Supplementary Information 4: Protein storage and quantification

Glycerol was added to freshly purified protein solution to a final concentration of 25%. Small aliquots were then flash frozen to liquid nitrogen temperature and stored at -80 °C until they were thawed and used immediately for the next experiment.

Short dsDNA containing 2.5 repeats of human telomeric sequence (2.5Telo) (Supplementary Figure 15b) was used to confirm the activity of the purified TRF2 proteins. 2.5Telo was obtained by annealing two complimentary oligos as described in the previous section (DNA constructs). DNA binding activity of the proteins was confirmed by EMSA (Supplementary Figure 15e). Different amounts of the TRF2 proteins were incubated with 2.5Telo (0.5 μM) at 4 °C for 30 min. in a 10 μL volume containing 25 mM HEPES-Na pH 7.5, 50 mM NaCl, 1 mM DTT. After incubation, DNA gel loading dye (ThermoFisher Scientific) was added to the sample and resolved in a 5% polyacrylamide gel and visualized under UV by ethidium bromide staining. Supplementary Supplementary Figure 15e shows the binding of TRF2 proteins to the 2.5Telo DNA .

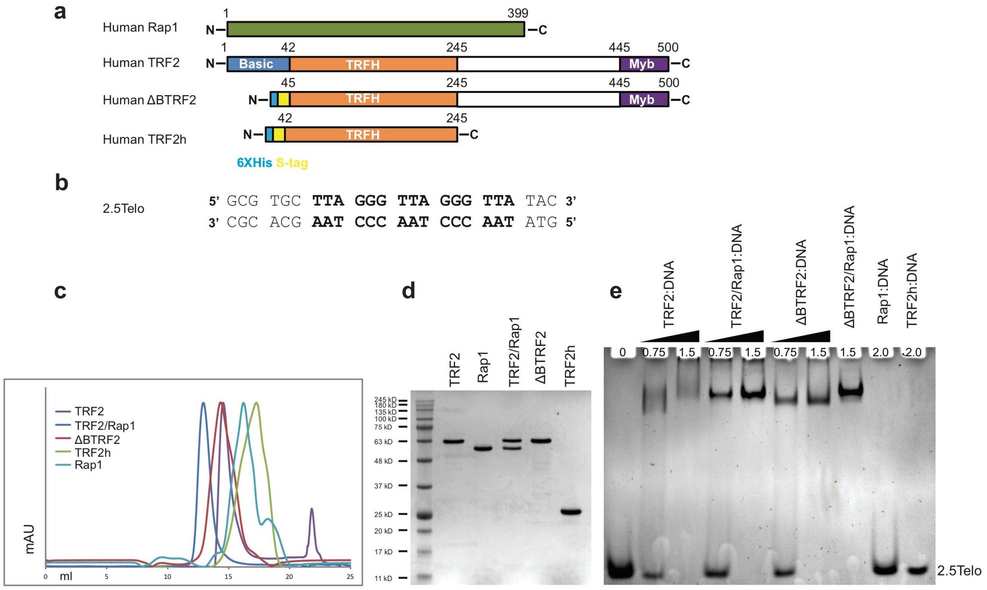

Supplementary Figure 15. hTRF2 purification and binding to telomeric DNA a Schematic representation of human Rap1 and human TRF2 protein constructs used in this study. **b** The sequence of the two-and-half repeats of telomeric DNA (2.5telo) used in EMSA (E) to confirm the activity of the proteins. **c** Elution profiles on Superose 6 10/300 GL column of bacterially expressed and purified human Rap1 and human TRF2 constructs used in this study. The data has been plotted after normalization. **d** 12% SDS-PAGE/Coomasie blue stain of the purified hTRF2, hRap1, hTRF2/Rap1, hΔBTRF2 and hTRF2h proteins concentrated from the Superose 6 10/300 GL column. **e** 5% Native gel showing the binding of TRF2, TRF2/Rap1, ΔBTRF2, ΔBTRF2/Rap1, Rap1 and TRF2h proteins to telomeric DNA (2.5Telo). The gel images in panel d & e are uncropped.

Supplementary References

---

[1] S. Le, M. Yao, J. Chen, A. K. Efremov, S. Azimi, and J. Yan, Nucleic Acids Research **43**, e113 (2015).

[2] N. Fouché, A. J. Cesare, S. Willcox, S. Özgür, S. A. Compton, and J. D. Griffith, Journal of Biological Chemistry (2006).
