# Supplementary material for: Exploring TRF2-Dependent DNA Distortion Through Single-DNA Manipulation Studies
Source: Commun Biol. 2024 Feb 3;7:148. doi: 10.1038/s42003-024-05838-x (PMC10838314; doi:10.1038/s42003-024-05838-x)
Supplement: Supplementary file 3 — Description of Additional Supplementary Files [file 42003_2024_5838_MOESM3_ESM.pdf]

Description of Additional Supplementary Files

File name: Supplementary Data

Description: The source data for the graphs in the main figures.
